# Extraction of phytochemicals from the pomegranate (*Punica granatum* L., *Punicaceae*) by reverse iontophoresis†

**DOI:** 10.1039/d3ra01242e

**Published:** 2023-04-11

**Authors:** Kieran Moore, Shaun B. Reeksting, Vimal Nair, Steve T. Pannakal, Nita Roy, Joan Eilstein, Sébastien Grégoire, M. Begoña Delgado-Charro, Richard H. Guy

**Affiliations:** a Department of Life Sciences, University of Bath UK prsbd@bath.ac.uk; b Agilent Technologies LDA UK 5500 Lakeside Cheshire UK; c Advanced Research, L'Oréal Research and Innovation India Bangalore India; d L'Oréal Aulnay-sous-Bois France

## Abstract

Plant metabolic profiling can provide a wealth of information regarding the biochemical status of the organism, but sample acquisition typically requires an invasive and/or destructive extraction process. Reverse iontophoresis (RI) imposes a small electric field across a biological membrane to substantially enhance the transport of charged and polar compounds and has been employed, in particular, to extract biomarkers of interest across human skin. The objective of this work was to examine the capability of RI to sample phytochemicals in a minimally invasive fashion *in fructo* (*i.e.*, from the intact fruit). RI was principally used to extract a model, bioactive compound – specifically, ellagic acid – from the fruit peel of *Punica granatum* L. The RI sampling protocol was refined using isolated peel, and a number of experimental factors were examined and optimised, including preparation of the peel samples, the current intensity applied and the pH of the medium into which samples were collected. The most favourable conditions (3 mA current for a period of 1 hour, into a buffer at pH 7.4) were then applied to the successful RI extraction of ellagic acid from intact pomegranates. Multiple additional phytochemicals were also extracted and identified by liquid chromatography with tandem mass spectrometry (LC-MS/MS). A successful proof-of-concept has been achieved, demonstrating the capability to non-destructively extract phytochemicals of interest from intact fruit.

## Abstract 2

Reverse iontophoresis facilitated the extraction of phytochemicals from intact pomegranates in a non-destructive manner. These phytochemicals were subsequently identified using liquid chromatography coupled to untargeted mass spectrometry (LC-MS).

## Graphical abstract

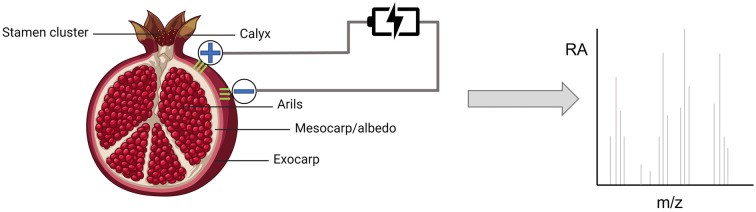

## Introduction

The plant kingdom provides a rich source of low molecular weight phytochemicals (<1500 Da) with each species “hosting” as many as 5000 metabolites – comprising, broadly speaking, primary and secondary metabolites and phytohormones – which fuel the biochemical reactions required for the survival and propagation of the organism.^1,2^ Characterisation of this plethora of chemicals has advanced significantly over recent years with respect to increasing sophistication, in terms of sensitivity, specificity, speed and high-throughput capabilities, of analytical and data processing platforms.^2–5^ As a result, exploitation of the vast array of compounds available, whether as nutrition supplements, ingredients in cosmetics and personal care products, or as potential therapeutics, represents an intense field of research and development activity.

Phytochemical characterisation requires processing of the plant biomass to acquire suitable samples (containing sufficient quantities of the target chemicals) for analysis. The workflow required, however, is often resource and labour-intensive and invariably demands the liberal use of environmentally-unfriendly, organic solvent extraction processes.^6^ As a result, sample preparation involves partial or complete destruction of the biomass and risks unintentional modification or degradation of the chemicals of interest.^6,7^

A preferred approach would involve minimally invasive sample extraction and preparation and offer *in situ* versatility, for example, to track biomarkers of fruit ripening without the need to harvest. In previous work, the application of reverse iontophoresis as a tool with which to extract plant phytochemicals from the intact leaves of *Ocimum basilicum* was demonstrated.^8,9^ The minimally invasive extraction, subsequently combined with sensitive ion chromatography-mass spectrometric analysis, permitted multiple phytochemicals to be easily detected, including organic acids, sugars and adenosine triphosphate.

Iontophoresis is an established technique that enhances the transport of (primarily) small, charged and/or polar molecules across biological membranes.^10^ Although used most often to improve drug delivery into and through the skin, so-called “reverse iontophoresis” (RI) has also been applied to the minimally invasive extraction of biomarkers from the interstitial fluid bathing the epidermis. The latter has been demonstrated *inter alia* both for drug monitoring (*e.g.*, lithium^11^) and for continuous tracking of glucose levels *in vivo* in man.^12^

In RI, a constant current – typically, on the order of 1 mA – is passed between positively and negatively charged electrodes (anode and cathode, respectively) immersed in electrolyte solutions on the biomembrane surface. The electric field induces the electromigration of positive cations towards the cathode and negative anions towards the anode.^13^ If the biomembrane supports a net charge, such as the skin which is negatively-charged at physiological pH, there is an induced electroosmotic flow in the direction of counterion movement which further enhances the electromigration and also enables the increased transport of polar, water-soluble compounds (such as glucose and zwitterions).^14,15^

To further validate the use of RI to extract phytochemicals minimally invasively from the biomass, the fruit of *Punica granatum* L., *Punicaceae* was selected. Pomegranate peel comprises a thick, inner albedo layer, which is white and spongy, and an outer exocarp, which is smooth and covered by a cuticle. The arils in the central part of the fruit are edible and contain the seeds and juice.^16,17^ The peel is known to be a rich source of bioactive compounds^16,18^ as reflected by the increasing global demand for pomegranate extracts believed to be beneficial to human health.^16,19–21^ This work also targets the increased interest in accurately monitoring the concentrations of specific compounds to better inform the timing of harvesting so that the yield of these phytochemicals may be maximised.^22^ Finally, the fruit of *Punica granatum* provides a different technical challenge, as a morphologically complex fruit, compared to the leaf of the basil plant, and offers, therefore, a test of RI's versatility as a novel monitoring platform of the biomass.

In the research presented here, a key first step was to identify and select a relevant bioactive compound for RI extraction. A number of experimental variables were then examined *in vitro* to optimise the conditions for reproducible and efficient extraction. Finally, the procedure was adapted and further refined to demonstrate the capability of RI to sample phytochemicals from intact pomegranates *in fructo* in a minimally invasive manner.

## Experimental

### Materials and methods

#### Chemicals

Analytical standards, phosphate buffer constituents (monosodium phosphate and disodium phosphate), sodium chloride, paracetamol, as well as organic solvents and water for chromatographic separation (LC-MS grade) were purchased from Merck UK (Poole, UK). Formic acid was from Fluka (NC, USA). De-ionised water (resistivity ≥ 18 MΩ cm) was obtained by filtering through a Milli-Q® system (Bedford, MA, USA).

#### Pomegranates

Fruits were purchased from a local Sainsbury's supermarket (Bath, UK) not more than one day prior to experiment. The cultivar (and origin) of the fruits were: Hicaz (Turkey), Aco (South Africa), and Wonderful (Peru). An unknown variety of Israeli origin was also used. Procurement was subject to availability; no attempt was made to control the cultivar or origin of the pomegranates used.

#### Electrodes

Materials for electrode preparation were obtained from Merck UK (Poole, UK). Silver/silver chloride cathodes were prepared by dipping silver wire (0.5 mm diameter) in molten silver chloride and were then ready for immediate use. To make the anodes, the silver chloride-dipped silver wire acting as an anode and a platinum wire (0.25 mm diameter), acting as cathode, were immersed in a 50 mM solution of sodium chloride and a 0.2 mA current was passed between them for ∼72 hours.

#### *In vitro* apparatus

**Fig. 1:**
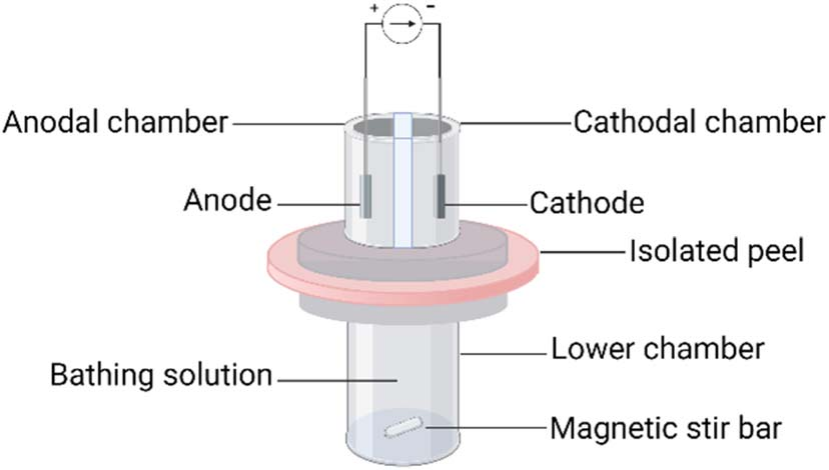
Schematic diagram of the 3-chamber reverse iontophoresis cell. Created with BioRender.com.

Peel was isolated by first dissecting the intact fruit into six spherical wedges and then using a sharp knife to remove the seeds. The albedo was separated so that an exocarp of ∼2 mm thickness remained. The exocarp was cut into circles of appropriate diameter and mounted in a previously described three-chamber iontophoresis cell,^23^ illustrated in Fig. 1. The outer surface of the pomegranate peel was oriented towards the electrode chambers (1 mL volume and 0.8 cm^2^ of peel exposed in each). The electrode solutions and that in the lower chamber (6 mL volume) comprised a 100 mM buffer containing 60–100 mM sodium chloride which was sufficient to sustain the electrochemistry for the duration of the experiment. The electrode chambers were electrically isolated from one another by an intervening space.

#### Initial screen for extractable phytochemicals *in vitro*

Peel from pomegranates of Israeli origin (unknown cultivar) was used. The solution in the electrode and lower chambers was 100 mM phosphate buffer at pH 7.4 with 100 mM sodium chloride. For 3 hours, no current was applied between the anode and cathode, and only passive diffusion accounted for the release of any endogenous compounds from the peel. Then, a direct current of 1 mA (1.25 mA cm^−2^) was passed for 1 hour using a programmable constant-current source (Kepco, NY, USA) and finally passive, ‘post-iontophoretic’ diffusion resumed for a further 1 hour. Over this 5 hour period, the solutions in the electrode chambers were sampled at 0.5, 1, 2, 3, 3.5, 4, and 5 h by removal and replenishment (with fresh buffer) of their entire contents.

#### Passive extraction of ellagic acid *in vitro*

A first series of experiments, involving two Hicaz (Turkey) pomegranates, assessed the effects of two peel preparation procedures on the passive extraction of ellagic acid (ESI Fig. S1†). In the first approach, the intact pomegranate was drenched under cold running water for 5 minutes prior to removal of the peel and mounting it in the diffusion cells. In half of these samples, the outer surface was further rinsed with four 1 mL washes with distilled water (P1A) whilst there was no second rinse for the other half (P1B). In the second approach, the fruit was first bisected longitudinally; one half was then drenched as before (P2A), the other not (P2B), prior to removing the peel and mounting in the diffusion cells. The outer surface of all peel portions was then rinsed with four 1 mL washes.

Following these different procedures, the passive extraction of ellagic acid was assessed. The diffusion cell upper and lower chambers contained 100 mM phosphate buffer, with 60 mM sodium chloride, at pH 7.4. After equilibration of the peel with the lower chamber solution for 1 h, 1 mL of the buffer was introduced into the upper chambers and passive extraction began. Sampling of the entire contents of the upper chambers was performed at 0.5, 1 and 2 h.

Subsequently, the intra-fruit variation of ellagic acid in the peel was assessed in a more conventional manner. The peel of one unused spherical wedge (*i.e.*, not used for diffusion replicates) from each pomegranate was divided latitudinally into three parts (‘top’, ‘middle’, and ‘bottom’) which were desiccated in an oven at 45 °C for 48 h. Using a pestle and mortar, the peel was then reduced to a fine powder, up to 15 mg of which was weighed into an Eppendorf tube (Stevenage, UK); 4 mL of pure methanol were added, and the suspension was sonicated for 30 minutes without temperature control in an ultrasonic bath (Clifton SW3H, Nickel-Electro Ltd, Weston-Super-Mare, UK). After sonication, the suspension was filtered through a 0.45 μm RC filter (Sartorius AG, Göttingen, Germany) and then diluted 10-fold in a 1 : 1 methanol/water mixture before analysis by liquid chromatography-mass spectrometry (see below). The crude extraction procedure was performed in triplicate for each fruit.

#### Reverse iontophoretic extraction of ellagic acid *in vitro*: effects of current and pH

To explore the parameters most appropriate for the optimal reverse iontophoretic extraction of ellagic acid, attention was focussed on the pH of the solutions on either side of the peel and on the applied current. Experiments were conducted, therefore, with solutions buffered (using phosphate buffer containing 60 mM sodium chloride) at either pH 7.4 or 4.0 and currents of 2 or 3 mA. For the measurements at pH 4.0, Hicaz (Turkey) and Wonderful (Peru) pomegranates were used; at pH 7.4, only Hicaz (Turkey) pomegranates were studied. The peel was prepared according to P2B (see ESI Fig. S1 and S2†) based on results obtained from the passive extraction series of experiments which examined four washing protocols. Prior to current application, the peel was allowed to equilibrate with the solutions in the upper and lower chambers of the diffusion cell for 1 hour. These solutions were then discarded and replenished with fresh buffer immediately before a direct current was applied for 1 hour. RI was performed at pH 4.0 with 3 mA intensity, and at pH 7.4 with 2 mA on the peel of one fruit and with 3 mA on that of the other. Every 15 minutes, current was interrupted while the entire contents of the electrode chambers were sampled and replenished. Passive extraction experiments, in the absence of current, were undertaken as controls.

#### Assessment of electroosmosis in reverse iontophoresis across isolated peel

To determine whether electroosmosis contributed significantly to the reverse iontophoretic extraction of endogenous compounds from pomegranate peel, experiments were performed in which the buffer in the lower chamber also contained paracetamol at a concentration of 66 mM (10 mg mL^−1^). Paracetamol is uncharged at the pH values of interest (4.0 and 7.4) and has previously and successfully been employed as a marker for electroosmotic flow in experiments on mammalian skin.^24^ The peel from four Hicaz (Turkey) pomegranates was used and the extraction of paracetamol into the (upper) anode and cathode chambers of the iontophoresis cell was measured at both pH 4.0 and pH 7.4 (as above). This series of experiments employed a 0.2 mA current and a 4 hour extraction period. This intensity was selected because it is known to induce a measurable electroosmotic flow across mammalian skin; furthermore, the longer extraction period is sufficient to overcome the ‘warm-up’ period observed prior to establishment of a ‘steady-state’ electroosmotic flow.^25,26^ The electrode solutions were sampled and replenished each hour and paracetamol extraction was determined by high-performance liquid chromatography with UV detection.

#### Reverse iontophoretic extraction of endogenous phytochemicals *in fructo*

**Fig. 2:**
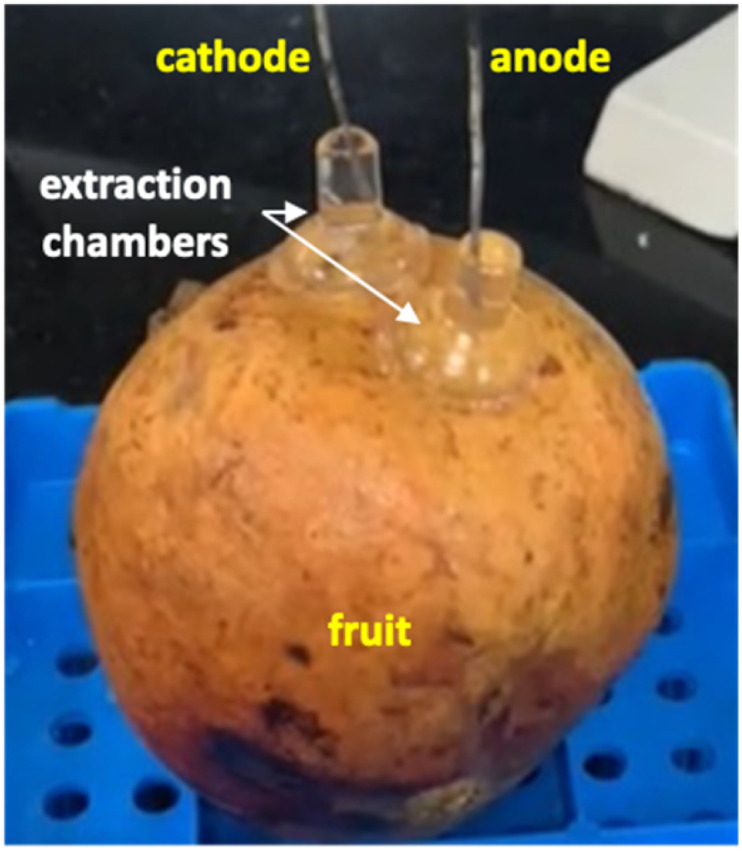
Illustration of the reverse iontophoresis experiment *in fructo*.

The pomegranates used were Hicaz (Turkey), Aco (South Africa) and Wonderful (Peru). Other than wiping the fruits with a clean, damp cloth, no other preparation was undertaken prior to the experiments. The experimental set up is shown in Fig. 2. Glass electrode chambers were positioned on the fruit surface, and a watertight seal was achieved using a layer of silicone grease. The exposed area was 0.8 cm^2^ in each chamber which were filled with 1 mL of 100 mM pH 7.4 PBS containing 60 mM sodium chloride. A current of 3 mA was passed between the electrodes for (initially) 30 minutes; samples were taken at 15 minutes and at current termination by removal and replenishment of the entire contents of the chambers. Data obtained from the first fruit under study were used to inform the prolongation of the sampling duration to 2 hours, with samples to be taken at 1 hour intervals. The concentration of sodium chloride was increased to 142 mM to account for the extended duration of anodal electrochemistry required. Replicate experiments were performed on the same fruit (using new cells, electrodes, buffer solutions) by rotating the fruit through ∼40° along its longitudinal axis and re-applying current as before. The approach enabled efficient data generation data from each fruit (and allowed intra-fruit variation to be addressed) and ensured that sampling sites were of a sufficient distance apart that the measurements could be considered completely independent of one another.

As a point of reference, the level of ellagic acid in the peel was determined after RI. The entire peel of the fruit was desiccated, weighed, and sonicated as described previously.

#### Liquid chromatography-mass spectrometric (LC-MS) analysis

Sample analyses were performed using an Agilent QTOF 6545 with Jetstream ESI spray source coupled to an Agilent 1260 Infinity II Quaternary Pump UHPLC (Agilent Technologies, Santa Clara, USA) with 1260 autosampler, column oven compartment and variable wavelength detector. The MS was operated in all-ions mode with scan range 50–1700 *m*/*z*, 20 000 : 1 resolution and was calibrated using a reference introduced from the independent ESI reference sprayer.

Samples from the *in vitro* experiments were injected in negative ionisation mode onto a reverse-phase InfinityLab Poroshell 120 EC-C18 (3.0 × 50 mm, 2.7 μm) column (Agilent Technologies, Santa Clara, USA) using 0.1% (v/v) formic acid in water (mobile phase A) and in methanol (mobile phase B). Accurate mass measurements are reported to four decimal places. For improved retention of amino acids, samples from the experiments *in fructo* were subjected to further LC-MS analyses using a HILIC column (Agilent InfinityLab Poroshell 120 HILIC-Z; dimensions, 2.1 × 100 mm, 2.7 μm). Details of the gradient schedules are given in ESI Table S1.† LC-MS operational conditions are in ESI Table S2.†

#### Identification of phytochemicals

Agilent Qualitative Analysis 10.0 software enabled compound searching of sample spectral results against a METLIN database of >80 000 compound entries. After the *in vitro* RI screening experiment, data generated from the injection of one anodal and one cathodal sample were interrogated using the ‘Find by Formula’ mining algorithm which returned molecular ions within 5 ppm of the reference library matches. The mass spectra of these ‘hits’ were exported to a personal compound database and library (PCDL). Where the hit target scores were >99, but were not found in the METLIN database, mass spectra were imported from other sources to find matches. The PCDL was used as a much smaller database for ‘qualifying’ hits for the rest of the samples. Qualification of molecular ions was performed using the accurate mass measurement of at least two fragment ions present in the MS/MS library spectra with a coelution score of >75. Relative quantification using the integrated areas of the extracted-ion chromatograms (EIC) was performed in Agilent Quantitative Analysis 10.0. The process was repeated after the *in fructo* experiments, with the additional interrogation of spectra generated subsequent to LC-MS injection using the HILIC chromatographic separation. Where available, analytical reference standards were used for further verification. Only qualified hits (*i.e.*, two co-eluting fragment ions) are reported.

Ellagic acid was quantified in negative ionisation mode after separation using the InfinityLab Poroshell 120 EC-C18 column. The retention time was confirmed to be 6.6 minutes with an analytical reference standard. Quantification in *in fructo* experiments used the integrated area of the ellagic acid EIC (4–300 ng mL^−1^ linear calibration range; *r*^2^ ≥ 0.99). For samples generated *in fructo* from Aco and Wonderful pomegranates, [^13^C_12_]-labelled ellagic acid (Alsachim, Illkirch-Graffenstaden, France) was used as an internal standard (IS). The IS was diluted to 10 μg mL^−1^ in acetonitrile, and 20 μL aliquots were added directly to 0.5 mL of sample before injection (20 ng mL^−1^ to 1.9 μg mL^−1^ linear calibration range; *r*^2^ ≥ 0.99).

#### Data analysis and statistics

Unless otherwise stated, the results are reported as arithmetic means (±standard deviation (SD)). Anodal, cathodal, and passive quantities of extracted ellagic acid were determined directly or, for uncalibrated extracted phytochemicals, relatively from the EIC response measured. From these results, extraction fluxes at each sampling interval could then be deduced (units: in μg h^−1^ or EIC response per h, respectively). Statistical analyses were performed using GraphPad Prism Version 9.0 software (San Diego, CA, USA) and the level of statistical significance was set at *α* ≤ 0.05.

## Results and discussion

### *In vitro* experiments

#### Initial screen for extractable phytochemicals

**Fig. 3:**
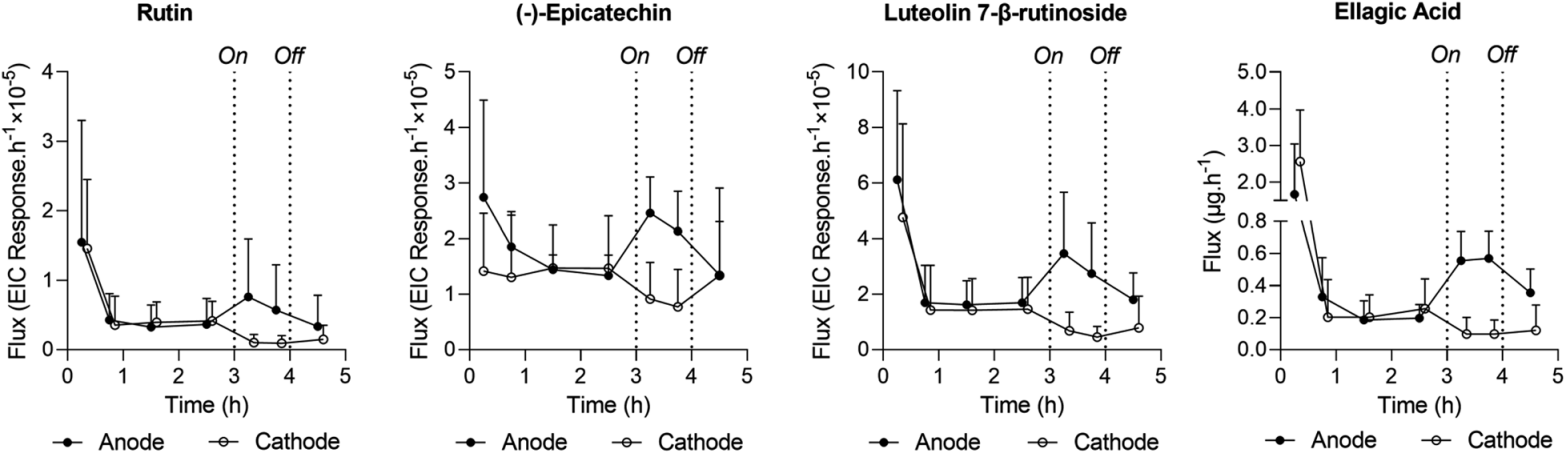
Extraction fluxes (plotted at the midpoint of the interval) of selected qualified phytochemicals as a function of time at the anode (closed circles; *n* ≥ 4) and cathode (open circles; *n* ≥ 4). Data have been slightly offset for clarity. Reported extracted ion responses represent integration (*i.e.*, peak areas) of the extracted ion chromatograms (EIC), therefore, representing peak area. The current application period is indicated by ‘on’ and ‘off’.

The initial screen generated several qualified hits, which are identified in ESI Table S3.† The extraction fluxes, into both the anodal and cathodal electrode chambers, of four illustrative phytochemicals as a function of time are presented in Fig. 3. The initially high values observed prior to application of the current are believed to be an artefact caused by the inadvertent exposure of the peel surface to juice from the fruit during the experimental preparation procedure. The passive, pre-iontophoresis fluxes (plotted at 0.75–2.5 h) were then seen to be low and steady. Application of the current (the third hour), resulted in enhanced extraction of all four compounds towards to anode (positive electrode) – relative to passive transport – but inhibited towards the oppositely charged cathode. This result is consistent with the anticipated (at least partially) negative charges on these compounds at pH 7.4 (see ESI Table S3†). After termination of current flow, the fluxes into the upper chambers appear to reconverge towards the values measured immediately prior to RI. This final observation indicates that the peel retained its integrity and justified the exploration of higher intensities in subsequent experiments.

Of the qualified phytochemicals extracted by RI, ellagic acid, a bioactive compound of therapeutic and cosmetic interest,^19,27,28^ was selected for further study. As the ellagic acid EIC responses were able to be calibrated using an analytical reference standard, it was possible to gauge the efficiency of the short period of electrotransport in terms of the percentage of the compound present in the peel that was extracted during current application. The dry mass of the peel exposed in the anodal chamber was 52.4 (±15.4) mg; following the sonication procedure described in the Materials and methods section, the recovered concentration of ellagic acid per mass of dry peel was 1.67 (±0.55) μg mg^−1^. Given that it was found that RI extracted ∼2.5 μg, it follows that short period of current application was able to ‘capture’ about 3% of the total compound present.

#### Passive extraction of ellagic acid

While the conventional solvent extraction of pomegranate peel revealed some differences in the spatial distribution of ellagic acid concentration (ESI Table S4†), the variation was never more than a factor of 2. In terms of the first peel preparation procedures, the passive extraction of ellagic acid during the first 30 minutes was significantly higher when the peel was not rinsed before the experiment began (P1B) (ESI Fig. S2,† left panel). This finding confirmed the effect of inadvertent deposition of juice on the peel surface and supports the idea that the high initial fluxes reported in the screening experiment (Fig. 3) were artefactual.

The second approach was designed to mimic heavy rainfall; the idea being that, should prospective fruits be sampled *in situ*, the rain may displace ellagic acid residing in the outermost layers of the peel, leading to an apparent difference in extraction behaviour *vs.* a fruit less exposed to precipitation. Should this be the case, it makes sense that subsequent experiments should incorporate a drenching procedure in an attempt to ‘equalise’ the surface of each pomegranate. Results, however, show that pre-drenching the fruit before peel isolation (P2A) had no significant effect on the subsequent passive extraction of ellagic acid (ESI Fig. S2,† right panel).

Together, these results informed the decision that subsequent *in vitro* experiments would not require drenching but would involve the rinse step on isolated peel once secured in cells prior to initiation of the extraction protocol. Therefore, P2B was employed for all subsequent *in vitro* experiments.

#### Effects of pH and current on the reverse iontophoretic extraction of ellagic acid *in vitro*

The return of post-iontophoretic fluxes to pre-iontophoretic levels during the final hour of the screening experiment (Fig. 3) suggested that the integrity of the membrane was not compromised by the current conditions employed (1 mA for a duration of 1 hour). This justified the investigation of higher intensities for enhanced extraction efficiency.

**Fig. 4:**
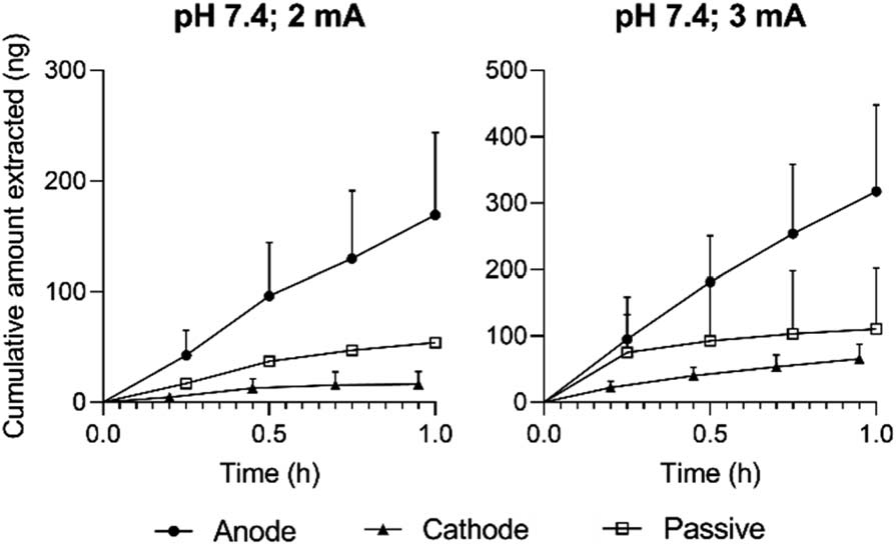
Cumulative reverse iontophoretic extraction of ellagic acid at pH 7.4 from isolated Hicaz pomegranate peel at currents of 2 or 3 mA. Extraction to anode (closed circles, *n* = 5), cathode (closed triangles, *n* = 5) and passively (open squares, *n* = 1 or 3) (mean ± SD). Data points are slightly offset for clarity.

The cumulative amounts of ellagic acid extracted from pomegranate peel (Hicaz, Turkey) by RI during 1 hour of either 2 or 3 mA current are shown in Fig. 4. Extraction (at both current levels) of ellagic acid to the anode was significantly greater (*p* < 0.05) than both that to the cathode and that achieved passively. When this experimental design was repeated with the electrode and lower chambers of the reverse iontophoretic cell containing a pH 4.0 buffer, there was no significant difference observed between the amounts of ellagic acid extracted with 3 mA current, and no significant improvement over passive transport was achieved either (ESI Fig. S3†). This difference in extraction behaviour is explained by the relative charge state of ellagic acid in each pH environment. Given the p*K*_a_ values of the ionisable groups are (p*K*_a_1__ = 5.4 and p*K*_a_2__ = 6.8 (ref. 29)), it is logical that extraction from a membrane buffered to pH 7.4 (in which ellagic acid is 99% ionised) should be more efficient than that from one fixed to pH 4.0 (imparting just ∼4% ionisation).

Approximately consistent with theory, anodal extraction at pH 7.4 was noticeably enhanced by not quite a factor of 2 when the current was increased to 3 mA. Extraction to the cathode was, as expected, inhibited relative to the passive no-current control, indicating that passage of a 3 mA current for 1 hour did not compromise peel integrity. As such, the favourable conditions taken forward for *in fructo* experimentation were a pH environment of 7.4 and an intensity of 3 mA.

#### Assessment of electroosmotic flow across pomegranate peel during iontophoresis

Given the sensitivity of enhanced reverse iontophoretic extraction to pH (as has been well-documented for mammalian skin^30,31^), experiments were performed to characterise the direction and importance of electroosmotic flow when a current was applied across pomegranate peel. In this approach, the reverse iontophoretic extraction of paracetamol into the electrode chambers from the lower compartment of the diffusion cell was assessed at pH 4.0 and pH 7.4. The results in ESI Fig. S4† demonstrate that the relative extraction to anode or cathode was close to unity over the 4 hour duration at either pH. In other words, the results were inconclusive as paracetamol did not appear to be preferentially extracted to either electrode, precluding the determination of a measurable electroosmotic flow.

#### *In fructo* experiments

**Fig. 5:**
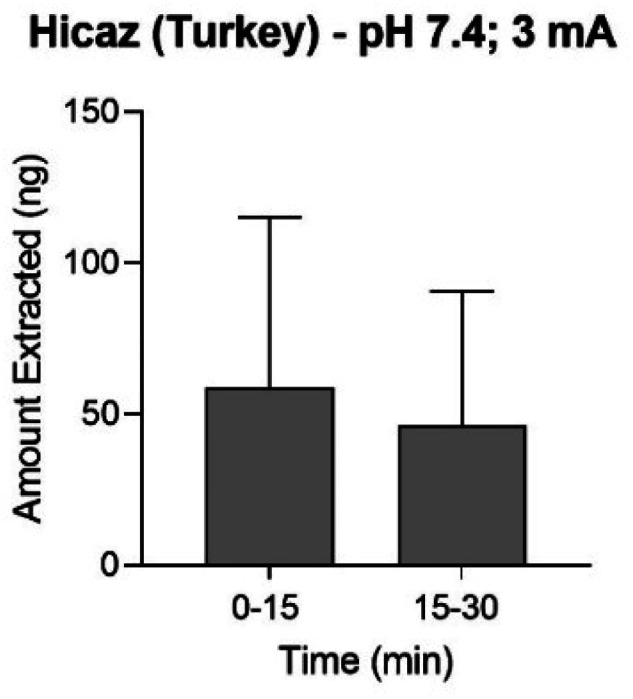
Anodal reverse iontophoretic extraction at pH 7.4 of ellagic acid from intact Hicaz pomegranates following two 15 minute periods of 3 mA current application (mean ± SD; *n* ≥ 4).

The favourable conditions deduced *in vitro* (pH 7.4 and 3 mA) were used to perform reverse iontophoretic extraction on the intact fruit. The initial range-finding experiment acquired samples at 15 minute intervals, over an abbreviated extraction period of 30 minutes in Hicaz (Turkey) pomegranate. Each sample contained approximately 50 ng of ellagic acid, corresponding to a flux of ∼0.2 μg h^−1^ (Fig. 5) which was closely aligned with *in vitro* results (Fig. 4). Ellagic acid was unquantifiable in the cathodal solution and no-current control experiment. Crude methanolic extraction was not performed for this fruit.

**Fig. 6:**
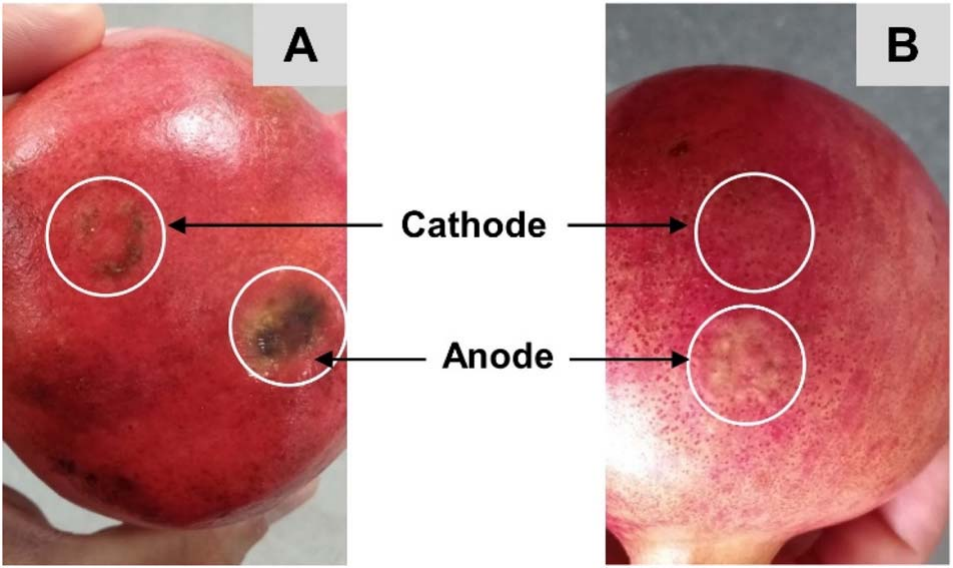
Discolouration at the sites of reverse iontophoresis on an Aco pomegranate after application of 3 mA current for 2 hours (A). Corresponding (lesser) impact on a Wonderful pomegranate subjected to the same treatment (B).

In subsequent experiments undertaken on Aco (South Africa) and Wonderful (Peru) pomegranates, the sampling time was extended to 1 hour and current was applied for a total of 2 hours. Ellagic acid was unquantifiable in the cathodal samples from the Peru (Wonderful) fruit but was detectable in those from the Aco (South Africa) variety; however, these EIC peak area responses were 1–2 orders of magnitude lower than the anodal samples. The amounts of ellagic acid iontophoretically extracted into the anodal chamber after the first and second hours of current passage were 2.1 (±1.0) μg and 1.3 (±1.1) μg, respectively, from the Aco fruit but these values were more than an order of magnitude lower in the Wonderful variety (ESI Table S5†). Notably, the solvent-extracted amounts of ellagic acid from the peels of the two types of pomegranate were within ∼3-fold of each other (ESI Table S5†). Some discoloration of the current-exposed sites of the fruits was observed and was more prominent for Aco than for Wonderful (Fig. 6A and B), though there was no indication that this caused any substantial increase in the amount of ellagic acid extracted in the second hour of RI when compared with the first. This finding also supports the (logical) assumption that ellagic acid is not the major charge-carrying ion during iontophoresis application. It is, of course, far more likely that this role will be performed by smaller, and more mobile, ions present in the fruit at higher concentrations, such as Ca^2+^ which is abundant in peel and arils.^32–34^

**Fig. 7:**
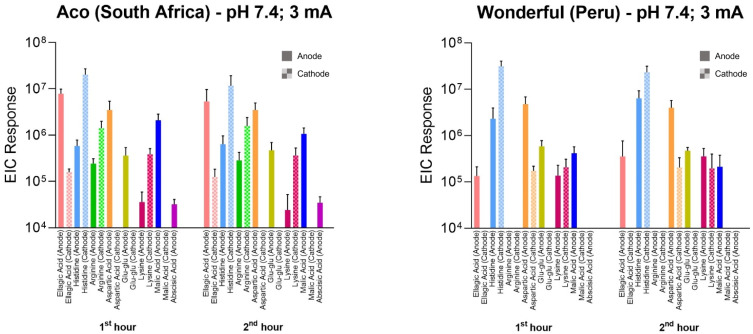
Reverse iontophoretic extraction of multiple phytochemicals to anode (filled bars) and cathode (stippled bars) from intact Aco and Wonderful pomegranates. The data are integrated peak areas from extracted ion chromatograms determined during the first and second hours of current passage (mean ± SD; *n* ≥ 4).

The opportunity was also taken to screen the RI samples (from anode and cathode chambers) for other extracted compounds from pomegranate peel. The results, in terms of integrated peak areas from extracted ion chromatograms (specifically, EIC peak area responses) from Aco and Wonderful fruits after the first and second hours of iontophoresis, are shown in Fig. 7; example chromatograms are in ESI Fig. S5 and S6.† Broader analysis of the extracts revealed a plethora of compounds: several charged species, both positive and negative, were identified, including lysine, arginine and histidine, glutamylglutamate, and aspartic, malic and abscisic acids. For both cultivars, the biomarker EIC peak area responses derived during the first and second hours of current application were compared at the relevant extraction electrode using paired *t*-tests. With the exception of histidine extracted from the Wonderful variety (for which significantly more was sampled during the first hour; *p* = 0.007), there were no statistical differences in the amounts extracted. In other words, the period of reverse iontophoresis did not appreciably deplete the biomarkers present and therefore no impact on their relative fluxes over time was observed. This finding supports the potential usefulness of the technique both to determine the metabolomic profile of a fruit and as a tool to assess more broadly the ripening process over time. Such applications, of course, will depend on further refinement of the RI protocol with respect to practicality, including the need to significantly shorten the duration of current application and the development of an easy-to-use, portable, and cost-effective device for use “in the field”. Other important requirements pertain to whether *in situ* analysis of the extracts is feasible, the extent to which (or the necessity for) a putative device to be calibrated against a relevant standard, and whether the liquid ‘receiving’ solutions can be replaced by more stable and easier-to-handle semi-solid alternatives.

## Conclusions

This research has demonstrated the feasibility of reverse iontophoresis to extract phytochemicals non-destructively from intact fruit. Proof-of-concept has been shown using the extraction of ellagic acid from pomegranates. Optimisation of the RI experimental parameters *in vitro* (specifically, the current applied and its duration, and the pH of the sampling medium into which ellagic acid was extracted) was undertaken before the concept was confirmed *in fructo*. Given the non-specific nature of RI, multiple other compounds were simultaneously extracted from the pomegranate peel, and a number of these were identified. Further work is clearly required to develop the findings from this study into a practical approach that can be used in the real-world to monitor, for example, the fruit ripening process.

## Conflicts of interest

There are no conflicts to declare.

## Supplementary Material

RA-013-D3RA01242E-s001

RA-013-D3RA01242E-s002

RA-013-D3RA01242E-s003

RA-013-D3RA01242E-s004

RA-013-D3RA01242E-s005

RA-013-D3RA01242E-s006

RA-013-D3RA01242E-s007
