# Supplementary figures and images for: Extraction of phytochemicals from the pomegranate (Punica granatum L., Punicaceae) by reverse iontophoresis
Source: RSC Adv. 2023 Apr 11;13(17):11261–8. doi: 10.1039/d3ra01242e (PMC10087384; doi:10.1039/d3ra01242e)

**Procedure 1  
(P1)**

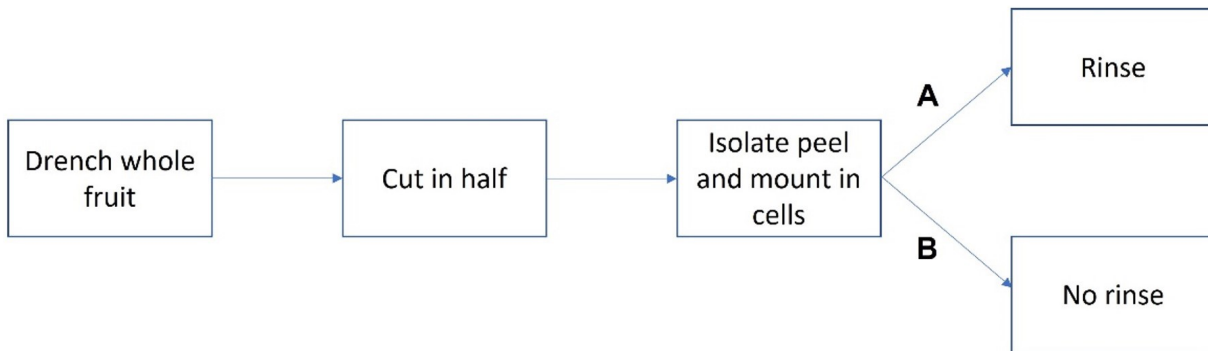

**Procedure 2  
(P2)**

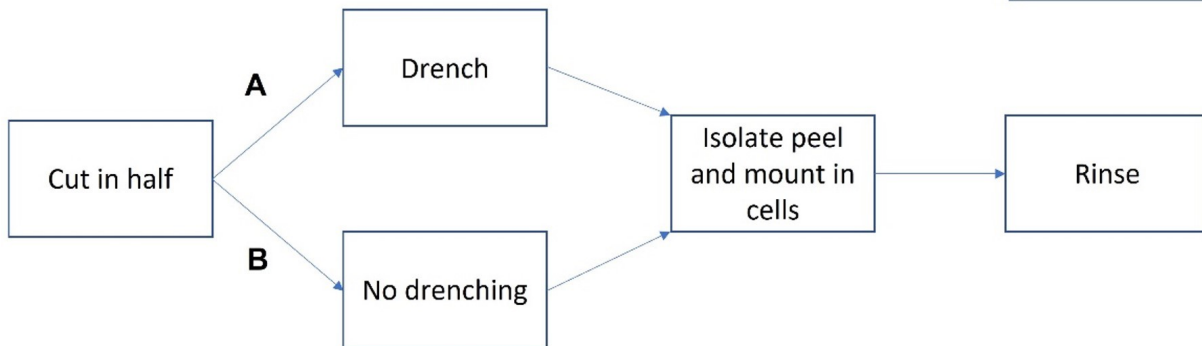

Supplement: RA-013-D3RA01242E-s001 [file RA-013-D3RA01242E-s001.pdf]

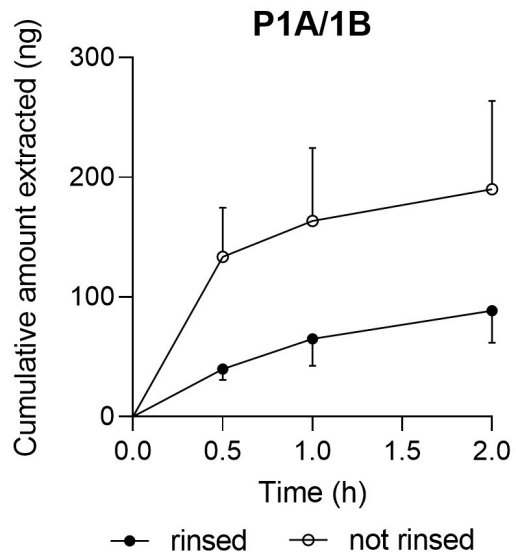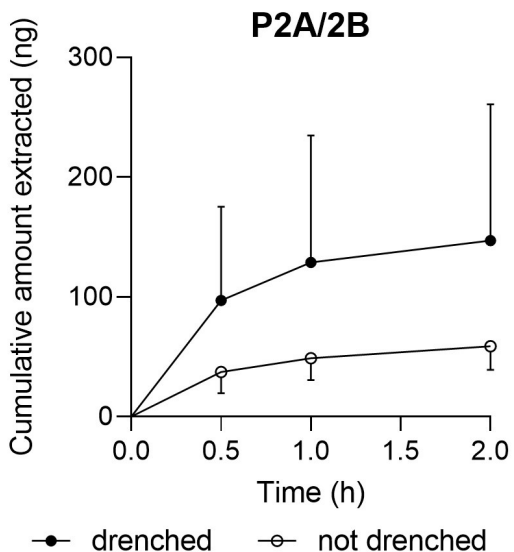

Supplement: RA-013-D3RA01242E-s002 [file RA-013-D3RA01242E-s002.pdf]

**Hicaz - pH 4.0; 3 mA**

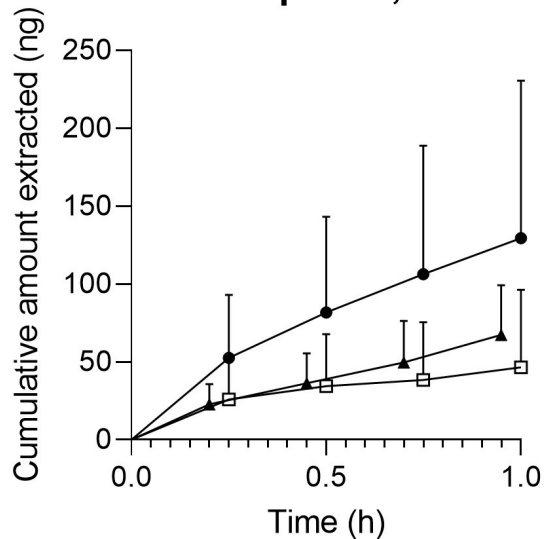

**Wonderful - pH 4.0; 3 mA**

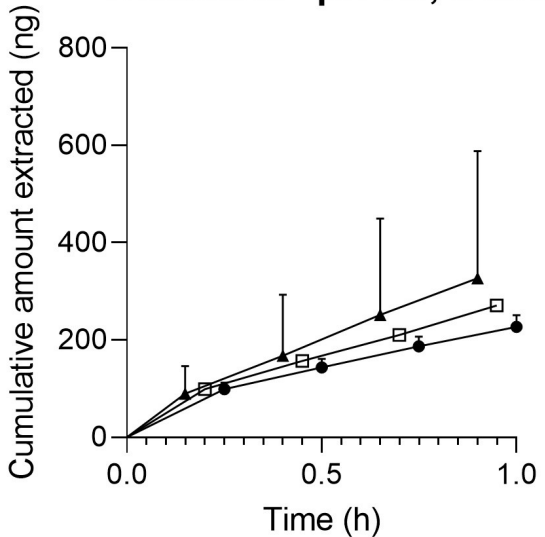

Supplement: RA-013-D3RA01242E-s003 [file RA-013-D3RA01242E-s003.pdf]

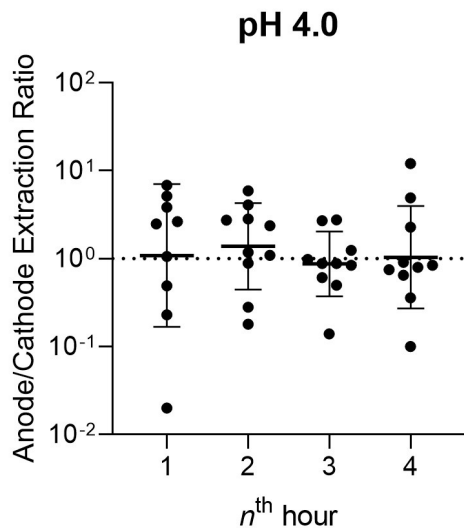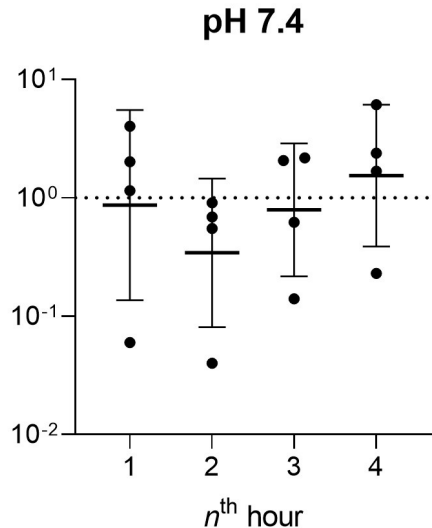

Supplement: RA-013-D3RA01242E-s004 [file RA-013-D3RA01242E-s004.pdf]

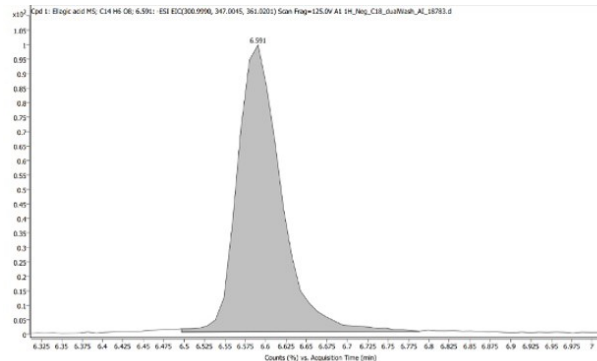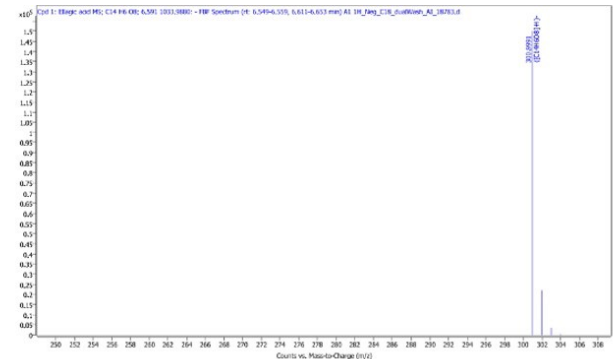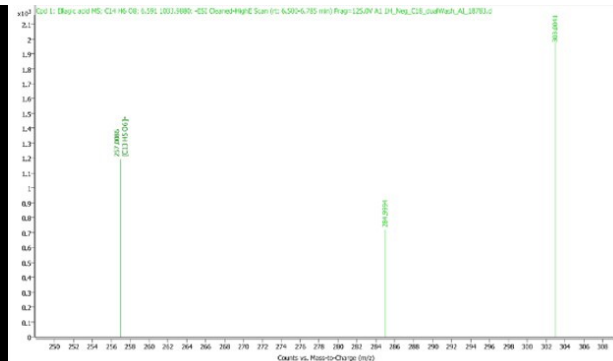

Supplement: RA-013-D3RA01242E-s005 [file RA-013-D3RA01242E-s005.pdf]

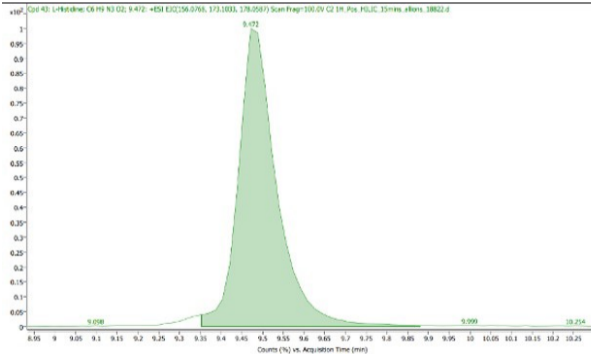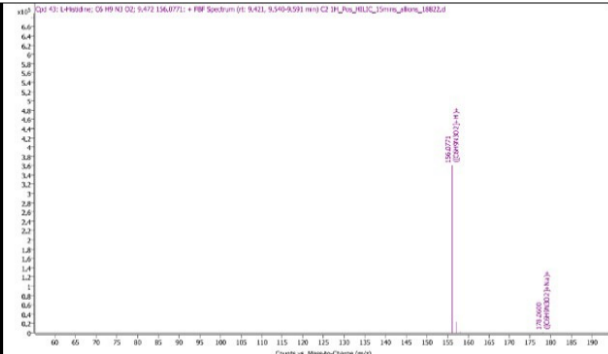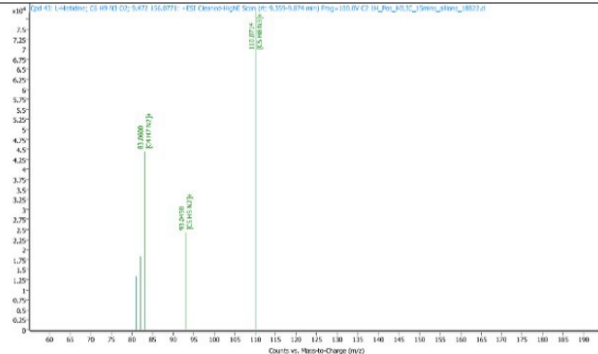

Supplement: RA-013-D3RA01242E-s006 [file RA-013-D3RA01242E-s006.pdf]
