# Supplementary material for: Extraction of phytochemicals from the pomegranate (Punica granatum L., Punicaceae) by reverse iontophoresis
Source: RSC Adv. 2023 Apr 11;13(17):11261–8. doi: 10.1039/d3ra01242e (PMC10087384; doi:10.1039/d3ra01242e)

Electronic Supplementary Material (ESI) for RSC Advances.
This journal is © The Royal Society of Chemistry 2023

Supplementary Information

Extraction of phytochemicals from the pomegranate (*Punica granatum L., Punicaceae*) by reverse iontophoresis

Kieran Moore [a*], Shaun Reeksting [b], Vimal Nair [c], Steve Pannakal [c], Nita Roy [c], Joan Eilstein [c], Sébastien Grégoire [d], M. Begoña Delgado-Charro [a], Richard H. Guy [a].

[a]Department of Life Sciences, University of Bath, UK.
[b]Agilent Technologies LDA UK, 5500 Lakeside, Cheshire, UK.
[c]Advanced Research, L'Oréal Research and Innovation India, Bangalore, India.
[d]L'Oréal, Aulnay-sous-Bois, France.

Supplementary Table S1: Chromatographic separation schedules.

Time (min)	% B (C18)	%B (HILIC)
0.0	5	100
1.0	20	-
4.0	45	-
4.5	95	-
6.5	95	-
7.0	5	-
8.0	95	-
10.0	95	-
10.5	5	-
11.5	-	30
12.0	-	100
12.5	5	-
15.0	-	100

Supplementary Table S2: LC-MS operational conditions for the two analysis methods employed.

**Parameter**	**C18 method**	**HILIC method**
Injection volume (µL)	5	5
Mobile phase flow rate (mL/min)	0.4	0.5
Gas temperature (^{o}C)	250	300
Drying gas flow rate (L/min)	12	13
Nebulising gas pressure (bar)	3.1	2.1
Sheath gas temperature (^{o}C)	350	350
Sheath gas flow rate (L/min)	12	12
VCap voltage (V)	3500	1500
Fragmentor voltage (V)	125	100
Skimmer voltage (V)	45	40
MS/MS scan segment collision energies (eV)	0, 20, 40	0, 20, 40

Supplementary Table S3: Summary of mass spectrometric data for bioactive compounds generated from *ex vivo* and *in vivo* RI experiments with their relevant physicochemical parameters. Putative hits were qualified based on precursor ppm mass error (within 5 ppm) with at least two curated fragment ions within 10 ppm mass error.

RT (min)	HPLC column	Putative phytochemical name (CAS number)	pK_a(s)	Charge at pH 7.4	Metabolite Class	Molecular Formula	ES(-/+) theor. m/z	ES(-/+) found m/z	Adduct	m/z error (ppm)	MS/MS ES(-) fragment ions
0.87*	C18	Citric acid†<br>(320-77-4)	3.1<br>4.8<br>6.4 [a]	-3	Organic acid	$C_6H_8O_7$	191.0192	191.0199	$[M-H]^-$	3.7	57.0346<br>67.0189<br>85.0295<br>87.0088<br>111.0088<br>154.9986<br>173.0092
6.97	C18	Luteolin 7-β-rutinoside†§ (20633-84-5)	8.3 [b]	0	Flavonoid glycoside	$C_{27}H_{30}O_{15}$	593.1506	593.1513	$[M-H]^-$	1.2	227.0383<br>255.0302<br>284.0302<br>285.04<br>286.0427<br>327.0510
4.98	C18	(-)-Epicatechin† (490-46-0)	8.3<br>(1)	0	Flavonoid	$C_{15}H_{14}O_6$	289.0712	289.0718	$[M-H]^-$	2.1	109.0295<br>179.0350<br>203.0714<br>205.0506<br>245.0819
6.60	C18	Rutin†<br>(115888-40-9)	6.4 [b]	-1	Flavonoid glycoside	$C_{27}H_{30}O_{16}$	609.1456	609.1457	$[M-H]^-$	0.2	178.9986<br>300.0334<br>301.0412
6.70*	C18	Ellagic acid†‡§‖ (476-66-4)	5.4<br>6.8<br>(2)	-2	Tannin	$C_{14}H_6O_8$	300.9984	300.9987	$[M-H]^-$	1.0	257.0086<br>284.9994
9.49*	HILIC	Histidine§‖<br>(71-00-1)	1.8<br>6.0<br>9.2 [a]	0	Amino acid	$C_6H_9N_3O_2$	156.0773	156.0772	$[M+H]^+$	-0.6	81.0448<br>82.0527<br>93.045<br>83.0601<br>110.0714
10.42*	HILIC	Lysine§	2.2	+1	Amino acid	$C_6H_{14}N_2O_2$	147.1133	147.1129	$[M+H]^+$	-2.7	56.0495

		(923-27-3)	9.0<br>10.5 [a]								84.0808
9.88[*]	HILIC	Arginine[§]<br>(74-79-3)	2.2<br>9.0<br>12.5 [a]	+1	Amino acid	$C_6H_{14}N_4O_2$	175.1195	175.1193	$[M+H]^+$	-1.1	60.0570<br>70.0651<br>116.0706<br>130.0975
8.88[*]	HILIC	Aspartic acid[§‖]<br>(56-84-8)	1.9<br>3.7<br>9.6 [a]	-1	Amino acid	$C_4H_7NO_4$	134.0450	134.0453	$[M+H]^+$	-2.2	46.0286<br>70.0288<br>74.0238<br>116.0341
9.36[*]	HILIC	Glutamylglutamic acid[§‖]<br>(3929-61-1)	2.2<br>4.3<br>9.7 [ad]	-2	Dipeptide	$C_{10}H_{16}N_2O_7$	277.1035	277.1041	$[M+H]^+$	2.2	84.0450<br>130.0496<br>148.06
0.81	C-18	Malic acid[§‖]<br>(6915-15-7)	3.5<br>5.0 [c]	-2	Organic acid	$C_4H_6O_5$	133.0137	133.0144	$[M-H]^-$	5.3	71.0139<br>72.9930<br>115.0033
7.13	C-18	Abscisic acid[§] (7773-56-0)	4.7 [b]	-1	Phytohormone	$C_{15}H_{20}O_4$	263.1283	263.1288	$[M-H]^-$	1.9	153.0919<br>203.1077<br>219.1386

[a] https://www.rsc.org/merck-index
[b] https://hmdb.ca/metabolites
[c] https://pubchem.ncbi.nlm.nih.gov
[d] pK_a values based on those of glutamic acid
[*]Verified with analytical reference standard
† RI extraction (isolated peel) confirmed from unknown cultivar (Israel)
‡ RI extraction *in fructo* confirmed from Hicaz (Turkey)
§ RI extraction *in fructo* confirmed from Aco (South Africa)
‖ RI extraction *in fructo* confirmed from Wonderful (Peru)

Supplementary Table S4: Intra-fruit variability in two Hicaz (Turkey) pomegranates

Peel section	Ellagic acid concentration in dry peel (µg/mg)	
	Experiment P1A & P1B	Experiment P2A & P2B
Top	2.62 (± 0.19)	1.53 (± 0.10)[b]
Middle	1.96 (± 0.22)[a,b]	1.57 (± 0.04)[b]
Bottom	2.72 (± 0.20)	1.94 (± 0.06)

[a] Significantly different to Top

[b] Significantly different to Bottom

Supplementary Table S5: Measured parameters from the reverse iontophoresis extraction of ellagic acid (EA) on intact pomegranates

Cultivar	% loss on drying	pH of dry peel suspension	Ellagic acid in dry peel (µg/mg)	EA extracted in 1st hr (ng)	EA extracted in 2nd hr (ng)
Aco (South Africa)	69	3.7	3.78 (± 1.70)	2060 (± 1001)	1321(± 1069)
Wonderful (Peru)	63	3.4	1.09 (± 0.04)	53 (± 33)	58 (± 32)

Supplementary Figure S1: Details of the four peel preparation procedures examined.

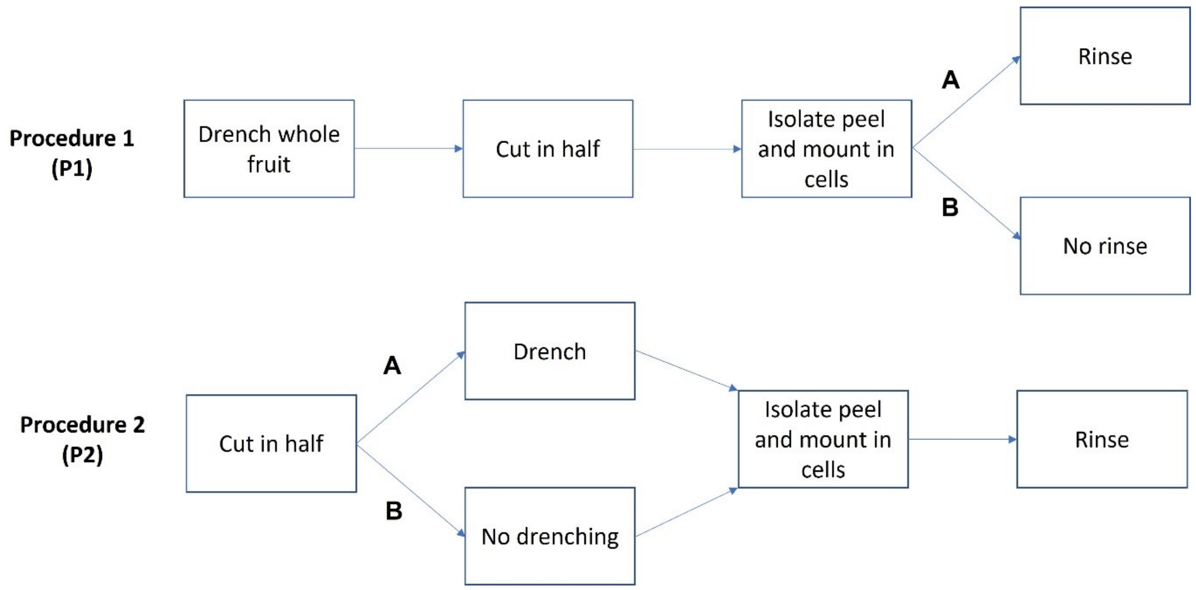

Supplementary Figure S2: Cumulative passive extraction of ellagic acid following [left panel] P1A and P1B, and [right panel] P2A and P2B (mean ± SD; n = 5 except for P2B for which n = 6).

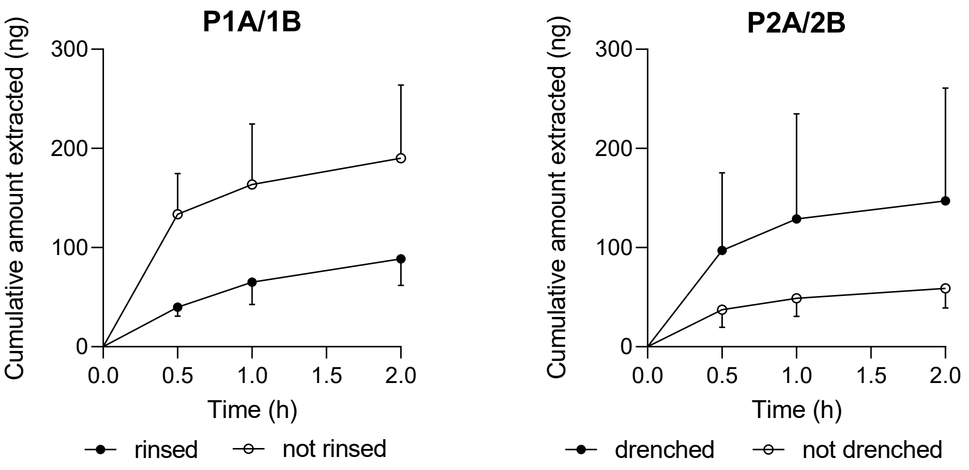

P1A/1B
Cumulative amount extracted (ng)
300
200
100
0
0.0
0.5
1.0
1.5
2.0
Time (h)
rinsed
not rinsed
P2A/2B
Cumulative amount extracted (ng)
300
200
100
0
0.0
0.5
1.0
1.5
2.0
Time (h)
drenched
not drenched

Supplementary Figure S3: Cumulative reverse iontophoretic extraction of ellagic acid at pH 4.0 from the isolated peels of Hicaz and Wonderful pomegranates at currents 3 mA current. Extraction to anode (closed circles, *n* = 4 or 5), cathode (closed triangles, *n* = 5) and passively (open squares, *n* = 1 or 3) (mean ± SD). Data points are slightly offset for clarity.

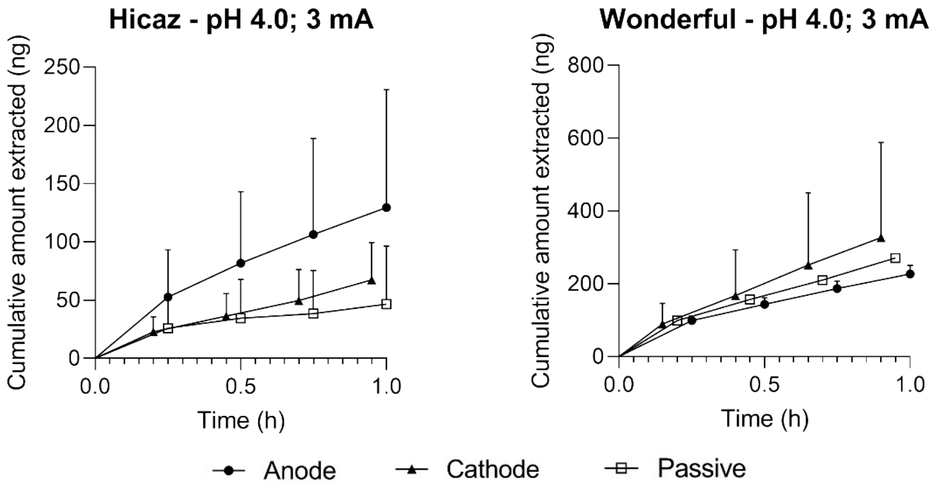

Supplementary Figure S4: Pooled anode-to-cathode extraction ratios of paracetamol as a function of the time of current (0.2 mA) application at pH 4.0 and pH 7.4 (geometric mean ± SD; *n* ≥ 9 and *n* = 5, respectively).

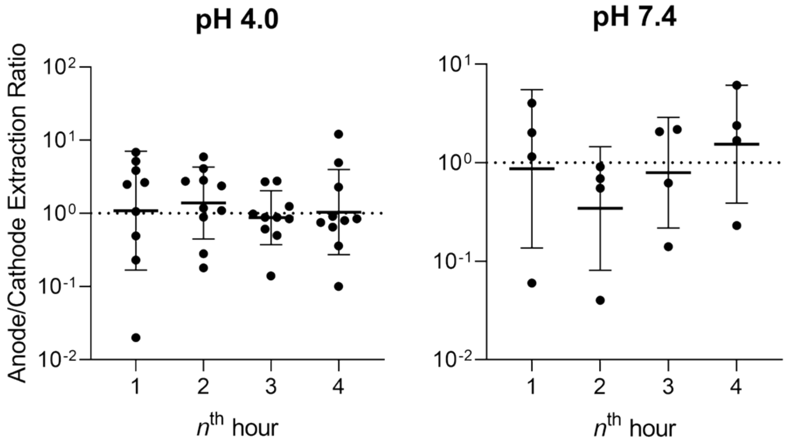

Supplementary Figure S5: Representative ellagic acid analysis of a RI extracted anodal sample from an intact Aco pomegranate *in fructo* after 1 h of current passage. Left panel - extracted ion chromatogram (EIC). Middle panel - molecular ion $[M-H]^-$ MS/MS spectrum. Right panel - MS/MS spectrum.

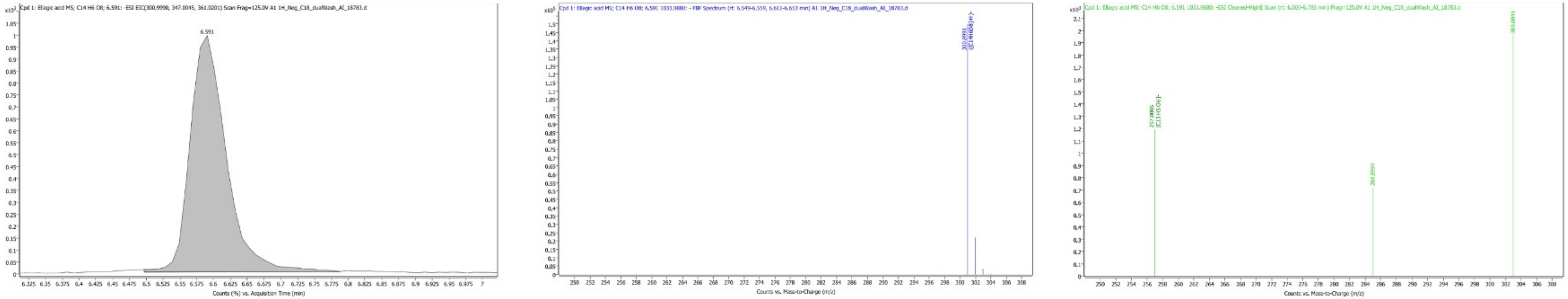

Supplementary Figure S6: Representative histidiine analysis of a RI extracted cathodal sample from an intact Aco pomegranate *in fructo* after 1 h of current passage. Left panel - extracted ion chromatogram (EIC). Middle panel - molecular ion $[M+H]^+$ MS/MS spectrum. Right panel - MS/MS spectrum.

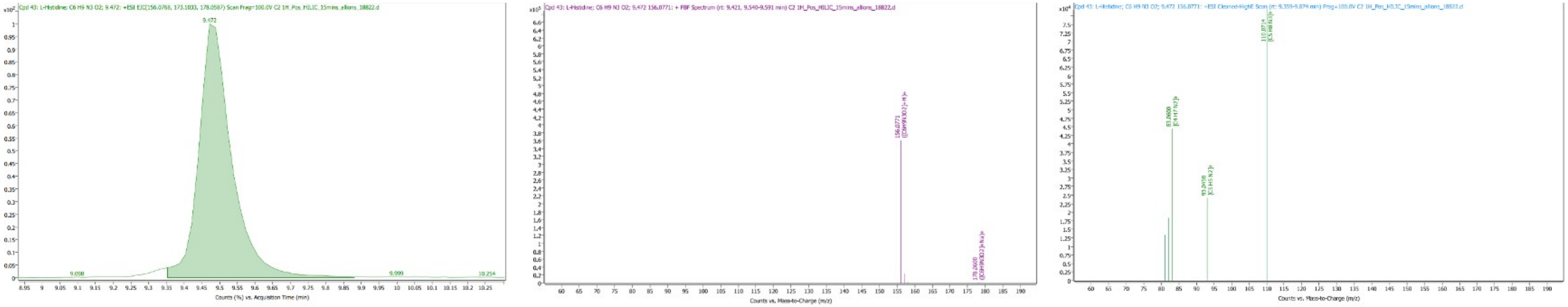

Supplement: RA-013-D3RA01242E-s007 [file RA-013-D3RA01242E-s007.pdf]
